# Rapamycin Pharmacokinetic and Pharmacodynamic Relationships in Osteosarcoma: A Comparative Oncology Study in Dogs

**DOI:** 10.1371/journal.pone.0011013

**Published:** 2010-06-08

**Authors:** Melissa C. Paoloni, Christina Mazcko, Elizabeth Fox, Timothy Fan, Susan Lana, William Kisseberth, David M. Vail, Kaylee Nuckolls, Tanasa Osborne, Samuel Yalkowsy, Daniel Gustafson, Yunkai Yu, Liang Cao, Chand Khanna

**Affiliations:** 1 Comparative Oncology Program, Center for Cancer Research, National Cancer Institute, Bethesda, Maryland, United States of America; 2 Division of Oncology, The Children's Hospital of Philadelphia, Philadelphia, Pennsylvania, United States of America; 3 University of Illinois, Urbana, Illinois, United States of America; 4 Colorado State University, Fort Collins, Colorado, United States of America; 5 Ohio State University, Columbus, Ohio, United States of America; 6 University of Wisconsin-Madison, Madison, Wisconsin, United States of America; 7 Tumor Metastasis and Biology Section, Center for Cancer Research, National Cancer Institute, Bethesda, Maryland, United States of America; 8 University of Arizona College of Pharmacy, Tucson, Arizona, United States of America; 9 Molecular Targets Core, Center for Cancer Research, National Cancer Institute, Bethesda, Maryland, United States of America; National Cancer Institute, United States of America

## Abstract

**Background:**

Signaling through the mTOR pathway contributes to growth, progression and chemoresistance of several cancers. Accordingly, inhibitors have been developed as potentially valuable therapeutics. Their optimal development requires consideration of dose, regimen, biomarkers and a rationale for their use in combination with other agents. Using the infrastructure of the Comparative Oncology Trials Consortium many of these complex questions were asked within a relevant population of dogs with osteosarcoma to inform the development of mTOR inhibitors for future use in pediatric osteosarcoma patients.

**Methodology/Principal Findings:**

This prospective dose escalation study of a parenteral formulation of rapamycin sought to define a safe, pharmacokinetically relevant, and pharmacodynamically active dose of rapamycin in dogs with appendicular osteosarcoma. Dogs entered into dose cohorts consisting of 3 dogs/cohort. Dogs underwent a pre-treatment tumor biopsy and collection of baseline PBMC. Dogs received a single intramuscular dose of rapamycin and underwent 48-hour whole blood pharmacokinetic sampling. Additionally, daily intramuscular doses of rapamycin were administered for 7 days with blood rapamycin trough levels collected on Day 8, 9 and 15. At Day 8 post-treatment collection of tumor and PBMC were obtained. No maximally tolerated dose of rapamycin was attained through escalation to the maximal planned dose of 0.08 mg/kg (2.5 mg/30kg dog). Pharmacokinetic analysis revealed a dose-dependent exposure. In all cohorts modulation of the mTOR pathway in tumor and PBMC (pS6RP/S6RP) was demonstrated. No change in pAKT/AKT was seen in tumor samples following rapamycin therapy.

**Conclusions/Significance:**

Rapamycin may be safely administered to dogs and can yield therapeutic exposures. Modulation pS6RP/S6RP in tumor tissue and PBMCs was not dependent on dose. Results from this study confirm that the dog may be included in the translational development of rapamycin and potentially other mTOR inhibitors. Ongoing studies of rapamycin in dogs will define optimal schedules for their use in cancer and evaluate the role of rapamycin use in the setting of minimal residual disease.

## Introduction

Signaling through the mTOR pathway has been linked to growth, progression and chemoresistance of several cancers [1], [2], [3], [4], [5], [6], [7], [8], [9], [10], [11]. Accordingly, agents that act against this pathway have been considered as potentially valuable therapeutics for cancer. Rapamycin, the originally described mTOR pathway inhibitor, is currently approved as an immunosuppressive agent used during preparatory and maintenance regimens for organ and bone marrow transplant patients. Preclinical studies of rapamycin in mice as well as recent data using novel and approved rapalogs (Ridaforolimus, Ariad; Temsirolimus, Wyeth) [12] in human patients suggest that mTOR blockade may be active in several cancers including sarcoma [2], [13], [14], [15], [16]. Based on responses in sarcomas, phase II/III clinical trials of rapalogs have been initiated in this patient population. The development of mTOR inhibitors as agents for sarcoma patients requires optimization of dose and regimen, defining informative biomarkers of effective exposure and activity, and rationale for their use in combination with existing or other novel drugs. An integrated and comparative approach that includes dogs with naturally occurring sarcoma may be uniquely suited to inform these development questions.

**Figure 1:**
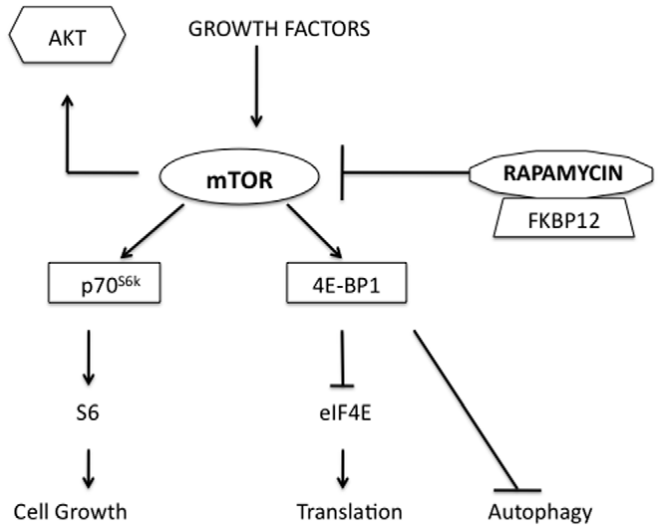
The mTOR pathway is integral in cell metabolism and protein translation in cancer. The mTOR pathway is the “nutrient sensor” of the cell and proximate targets of the pathway are responsible for both TOP and cap-dependent translation of proteins. Many of these proteins have been shown to be important in cancer progression, angiogenesis, autophagy and anti-apoptotic mechanisms. Rapamycin inhibits mTOR (via TORC1) following the formation of a complex with FKBP-12.

The mTOR pathway is the “nutrient sensor” of the cell and proximate targets of the pathway are responsible for both terminal oligopyrimidine (TOP) and cap-dependent translation of proteins (**Figure 1**) [17]. Many of these proteins have been shown to be important in cancer progression, angiogenesis, autophagy and anti-apoptotic mechanisms [3], [18], [19], [20]. Rapamycin inhibits mTOR (via TORC1) following the formation of a complex with FKBP-12 [17]. This results in decreased mTOR kinase activity, inhibited phosphorylation of downstream targets such as p70 ribosomal protein S6 kinase (S6RP) and 4E-binding protein (4E-BP1), and potentially suppression of ribosome biogenesis and protein translation [3], [17]. Interestingly, in some cancer histologies up-regulation of pAKT following mTOR inhibition has been seen both in preclinical models and in patients on receiving rapalogs in clinical trials. Since up-regulation of AKT can be predictor of chemoresistance and an aggressive phenotype this observation requires further investigation in a clinically relevant setting [21], [22].

Work by us and others have highlighted the importance of the mTOR pathway and the therapeutic benefit associated with mTOR inhibition in several cancers including pediatric sarcomas [2], [13], [14], [23]. Early phase clinical studies using rapamycin or rapamycin analogues (rapalogs) in pediatric sarcoma are currently underway. As discussed above the development of rapamycin and other rapalogs for use in pediatric osteosarcoma will require information on the optimal schedule and regimen for rapamycin, to understand the consequences of immunosuppression in patients, and the potential utility of rapamycin in the setting of minimal residual disease. Studies in dogs with osteosarcoma are well positioned to address these development concerns generating relevant translational data to inform the planned design of phase II/III pediatric rapalogs trials.

Although not rigorously reported in the literature, there has been a concern about a unique sensitivity of dogs to rapamycin [24], [25]. This sensitivity appears to include vasculitis (seen in most mammalian species), particularly manifested in the gastrointestinal tract following administration of varying doses of rapamycin [26], [27], [28], [29], [30]. Importantly pharmacokinetic (PK) analysis of rapamycin in dogs has not been reported. Defining exposure and modifying dose, schedule and/or route of administration may mitigate toxicity in dogs. Accordingly, before the dog could be integrated within the development path of mTOR inhibitors in osteosarcoma patients, preliminary studies with rapamycin in dogs were necessary.

The Comparative Oncology Trials Consortium (COTC) represents a large-scale collaborative effort of the NCI and extramural academic veterinary oncology programs [24]. Through the COTC, the rapid evaluation of early cancer drugs using biologically intensive trials has begun, utilizing the model system of companion animals (dogs) with cancer. The study's primary objective was to identify a safe, pharmacokinetically and pharmacodynamically relevant dose of rapamycin in tumor bearing dogs, so as to comfortably include the dog in future development studies. Results established that parenteral rapamycin was well tolerated in dogs at PK exposures that allow translation to human patients. All exposures of rapamycin evaluated resulted in downward modulation of phosphorylation of the mTOR target S6RP in tumors and PBMCs. Study data provide the basis to include the dog in the study of mTOR inhibitors as part of their development in pediatric osteosarcoma and other cancers. On-going efforts in dogs are underway to validate novel pharmacodynamic biomarkers of relevant clinical exposure to rapamycin, to assess the impact of immunosuppressive and non-immunosuppressive schedules of rapamycin in cancer, and to assess the activity of rapamycin in the minimal residual disease setting in osteosarcoma.

## Results

### Cell lines and *in vitro* inhibition with rapamycin

Little is known about the status of mTOR biology in canine osteosarcoma [31]. Studies done in preparation for this trial showed that components of the mTOR pathway, mTOR, p-S6, p-4EBP1 are expressed in canine osteosarcoma cell lines and primary tumors (data not shown). Using both western blot and electrochemiluminescence (ECL) analysis, rapamycin inhibits down-stream targets of mTOR in a dose-dependent fashion. (**Figure S1**) [31]. These data supported the credentials of canine osteosarcoma as a model for human osteosarcoma and more broadly as a solid tumor sensitive to mTOR inhibitor therapy.

### Study design and schedule

The study design was a dose escalation approach (**Table 1** **: Dose Escalation Cohorts and** **Table 2** **: Study Schedule**) to define relevant exposures of rapamycin and/or maximally tolerated dose (MTD) in dogs with appendicular osteosarcoma. All dogs underwent a pre-treatment tumor biopsy and collection of PBMC at study initiation. Dogs received a single intramuscular dose of rapamycin and underwent 48-hour PK whole blood collections. Dogs were then administered daily intramuscular doses of rapamycin for 7 consecutive days at fixed dose. All had post-treatment collection of tumor and PBMC. The defined study period was 15 days. Safety, tumoral pharmacodynamic (PD) modulation, correlation to surrogates (PBMC), and the relationship between PK and PD were study endpoints.

**Table 1:** Rapamycin Dose Escalation cohorts in dogs with osteosarcoma.

Dose Cohort	Rapamycin dose cohorts (7 day daily IM schedule	# of dogs in cohort	Median weight (kg)/cohort	Approximate rapamycin dose administered IM QD
1	0.01 mg/kg IM daily	3	43.2	0.35 mg
2	0.02 mg/kg IM daily	4	36	0.70 mg
3	0.04 mg/kg IM daily	3	35.6	1.4 mg
4	0.06 mg/kg IM daily	7	38.5	2.1 mg
5	0.08 mg/kg IM daily	4	32.5	2.8 mg

**Table 2:** Rapamycin Dose Escalation Study Schedule.

Action	Pre tx	Day <0	Day 0	Day 1	Day 8	Day 15
Patient Eligibility	X					
Tumor Biopsies		X			X	
Measurement of tumor burden (radiograph)		X			X	
Digital photo of tumor		X			X	
CBC/chemistry profile/UA			X		X	X
PBMC collection			X		X	X
Serum, plasma collection			X	X	X	X
Owner Assessment Form			X		X	X

### Dose Escalation Process and Patient Characteristics

Dogs were entered to rapamycin dose cohorts consisting of 3 dogs per cohort (n = 22 enrolled, n = 19 dogs completed study). Escalation through 5 planned dose cohorts was based on assessment of dose limiting toxicities (DLT) using VCOG modified-CTCAE convention [32]. Age (range, 3.7–11.9 years; median 7.9 years), sex (14 spayed females, 8 castrated males) and breed (5 mixed-breed and 17 purebred) were recorded for all dogs enrolled on study (**Table 3** **: Patient Characteristics**).

**Table 3:** Patient Characteristics.

Site	Patient	Dog's Sex	Age	Breed	Primary Disease Site	Stage of Disease	Cohort
CSU	0201	Spayed female	8.6	Rottweiler	Humerus	I	0.01 mg/kg IM daily
OSU	0601	Spayed female	5.1	Mixed Breed	Tibia	II	0.01 mg/kg IM daily
UW	0501	Castrated male	9.3	Weimaraner	Radius		0.01 mg/kg IM daily
OSU	0602	Castrated male	5.7	Great Pyrenees	Tibia	II	0.02 mg/kg IM daily
OSU	0603	Spayed female	9.1	Weimaraner	Humerus	II	0.02 mg/kg IM daily
OSU	0604	Castrated male	5.9	Saint Bernard	Radius	II	0.02 mg/kg IM daily
UIL	0701	Castrated male	8.8	Irish Setter	Humerus	IIB	0.02 mg/kg IM daily
OSU	0605	Spayed female	11.9	Mixed Breed	Humerus	II	0.04 mg/kg IM daily
UIL	0702	Spayed female	8	Boxer	Tibia	IIB	0.04 mg/kg IM daily
UIL	0703	Spayed female	8.2	Mixed Breed	Radius	IIB	0.04 mg/kg IM daily
CSU	0202	Spayed female	3.7	Great Pyrenees	Tibia	II	0.06 mg/kg IM daily
CSU	0203	Spayed female	7.1	Mixed Breed	Radius	II	0.06 mg/kg IM daily
CSU	0204	Spayed female	4.6	Great Dane	Radius	II	0.06 mg/kg IM daily
CSU	0205	Spayed female	8.7	Labrador Retriever	Humerus	II	0.06 mg/kg IM daily
OSU	0606	Spayed female	10.2	Rottweiler	Tibia		0.06 mg/kg IM daily
UIL	0704	Castrated male	6	Irish Wolfhound	Tibia	IIB	0.06 mg/kg IM daily
UIL	0705	Castrated male	3.9	Beagle	Humerus	IIA	0.06 mg/kg IM daily
CSU	0207	Castrated male	5.8	Rottweiler	Humerus	I	0.08 mg/kg IM daily
CSU	0208	Castrated male	11.7	Labrador Retriever	Radius	I	0.08 mg/kg IM daily
UIL	0706	Spayed female	6.1	Mixed Breed	Radius	IIB	0.08 mg/kg IM daily
UIL	0707	Spayed female	10.4	Greyhound	Femur	IIB	0.08 mg/kg IM daily
UIL	0708	Spayed female	7.8	Rottweiler	Femur	IIB	0.08 mg/kg IM daily

Site of enrollment: CSU = Colorado State University, UIL = University of Illinois, OSU = Ohio State University, UW = University of Wisconsin; WHO staging for canine osteosarcoma: Stage I = low grade primary tumor, A = intracompartmental tumor, B = extracompartmental tumor, Stage II = high grade primary tumor/no metastatic disease (N0 and M0), A = intracompartmental tumor (in marrow), B = extracompartmental tumor.

Stage III =  low or high grade primary tumor with metastatic disease (N1 or M1), A = intracompartmental tumor, B = extracompartmental tumor.

### Parenteral administration of rapamycin was well tolerated by tumor-bearing dogs

Hematologic and biochemical laboratory tests were collected at baseline (Day 0) and weekly (Day 8 and Day 15) to evaluate the safety of short-term rapamycin exposure. All data were reported by contemporaneous electronic reporting (C3D) such that adverse events were uniformly monitored, managed, and attributed within this multi-center COTC trial design. Escalation of rapamycin, from administered doses of 0.35mg/dog to 2.8mg/dog (IM, QD) was well tolerated and a maximally tolerated dose (MTD) was not defined (n = 19).

No unexpected adverse events were noted. Self-limiting and non-dose limiting toxicities (grade 1,2) that may have been attributable to rapamycin included vomiting (n = 2), diarrhea (n = 1), anorexia (n = 2) and thrombocytopenia (n = 1). There were two febrile episodes reported n = 1 at Day 7 (patient 0707) and n = 1 at Day 14 (patient 0204). The day 14 event was a result of a second occult neoplasm. The Day 7 event although mild (grade 1), was clinically relevant as it was concurrent with thrombocytopenia (grade 2). This event was believed to be an idiosyncratic post-operative reaction, and recovered without intervention. A first event death at Day 10, one-day post-operatively (patient 0704) was recorded. Necropsy of this case revealed cause of death to be congestive heart failure due to occult cardiomyopathy exacerbated by anesthesia and surgery. Although this event was not drug related, three additional dogs were entered into this dose cohort during the resolution of necropsy findings and attribution of the event (cohort 4). No additional toxicities were observed in this expanded cohort. There were no clinically significant neurological, respiratory, renal, or biochemical toxicities related to the treatment of the dogs with rapamycin. Two dogs withdrew from study during the week of drug administration, one due to intractable pain at the primary tumor site and one due to owner request. Neither immunosuppression nor surgical incision healing delays were reported during this short-term rapamycin treatment or through the post-treatment observation period (Day 15).

### Rapamycin administration in dogs with cancer resulted in systemic exposures similar to those seen in human patients

**Figure 2:**
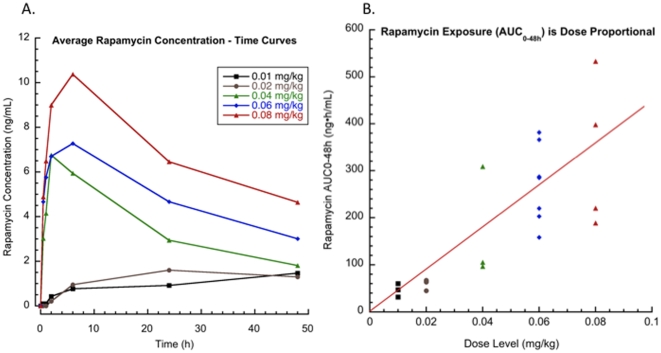
Rapamycin exposure in dogs with osteosarcoma is dose dependent. Serial rapamycin whole blood concentrations (ng/ml) were measured by HPLC with MS/MS detection for all dogs that completed study (n = 19). After a single parenteral dose of rapamycin, 7-point PK analysis (samples collected at 0, 30 minutes, 1,2, 6, 24 and 48 hours) was performed. Over the dose range studied, **A**. average concentration – time curves for each dose level, and **B**. rapamycin exposure (AUC_0–48h_) increased proportionally to dose.

**Figure 3:**
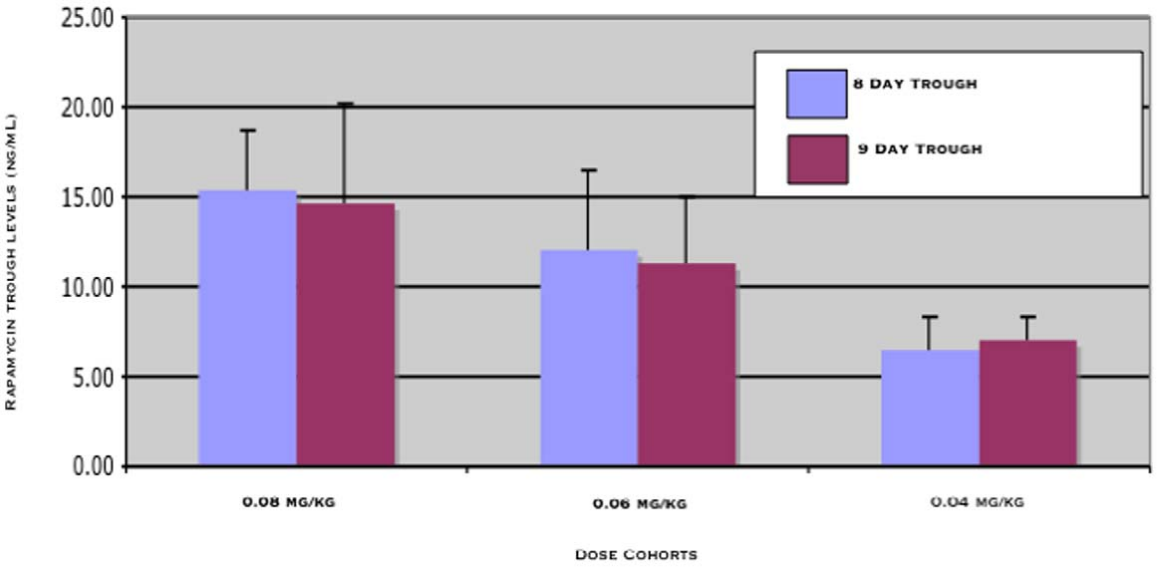
Translationally relevant exposures of rapamycin were achieved in dogs with cancer. In this study, dogs in the 4^th^ (0.06 mg/kg, approx 2.1 mg) and 5^th^ (0.08 mg/kg, approx 2.8 mg) dose cohorts had measurable trough levels ≥10 ng/ml. Trough concentrations in dogs with osteosarcoma after 7 and 8 days (C_D8_, C_D9_, ng/ml) of rapamycin treatment are similar to those intended for human cancer patients. Translationally relevant exposures of rapamycin are achievable in dogs with cancer, and support the use of the comparative approach in rapalog development.

Serial rapamycin whole blood concentrations (ng/ml) were measured for all dogs (0.01–0.08 mg/kg IM) on study by high-pressure liquid chromatography (HPLC) with tandem mass spectrometry (MS/MS) detection. Ten (10) samples per patient were collected at 0, 30 minutes, 1,2, 6, 24 and 48 hours, after the first rapamycin administration and then on days 8, 9 and 15 at 24-hour trough time points. The T_max_ ranged from 2–48hrs indicating that the absorption of rapamycin after IM injection in dogs was variable. Over the dose range studied, average concentration – time curves (**Figure 2A**) and systemic exposure (C_max_ and AUC_0–48h_, **Figure 2B**) increased proportionally to rapamycin dose (**Figure 2** and **Table 4**). The terminal half-life of rapamycin in dogs was greater than 60 hours. Steady state was not achieved after 8 days of exposure but PK analysis estimated time to steady state to be 12.5 days. Clearance could not be estimated because the sampling interval was shorter than the half-life. However, accumulation was evident at Day 15, seven days after cessation of therapy (C_D15_, ng/ml), therefore clearance may be lower than in humans [33]. At 0.06 mg/kg (approx 2.1 mg/day) and 0.08 mg/kg (approx 2.8 mg/day) dose levels, median trough concentrations on day 8 and 9 (C_D8_, C_D9_, ng/ml) were greater than 10 ng/mL, the putative trough target concentration in humans receiving rapamycin in the setting of transplantation [34]. In summary, translationally relevant exposures of rapamycin were achieved in dogs with cancer (**Figure 3**).

**Table 4:** Median (Range) Non-Compartmental Pharmacokinetic Parameters in dogs with cancer after 8 days of exposure to rapamycin.

Dose Level	N	AUC_0–48_	C_max_	T_max_	C_48h_	C_D8_	C_D9_	C_D15_	T_1/2_
mg/kg		ng•hr/mL	ng/mL	h	ng/mL	ng/mL	ng/mL	ng/mL	h
**0.01**	3	47.4 (31.9–60.2)	1.27 (0.79–2.92)	24 (24–48)	0.91 (0.56–2.92)	2.78 (1.77–3.39)	1.92 (0.5–3.14)	0.45 (0–0.89)	114.6
**0.02**	4	64.3 (44.9–67.3)	1.69 (1.21–1.82)	24	1.26 (1.11–1.55)	5.63 (5.4–5.65)	5.93 (4.42–7.15)	0.86 (0.78–0.95)	70.8 (23.2–88)
**0.04**	3	105.4 (96.9–308.9)	2.87 (2.39–18.2)	24 (2–24)	1.86 (1.38–2.19)	6.62 (3.18–9.72)	7.92 (3.2–10.1)	1.56 (0.32–1.91)	62.7 (17.6–88.6)
**0.06**	7	285.2 (158.2–381.8)	7.29 (3.87–17.8)	6 (2–24)	3.61 (2.11–4.73)	9.84 (6.49–17.6)	9.3 (7.9–16.6)	1.04 (0.87–3)	72.7 (58.2–95.8)
**0.08**	4	309.3 (188.9–533.0)	12.14 (5.36–21.1)	15 (2–24)	4.6 (3.94–5.45)	15.4 (15.1–19.6)	16.4 (8.65–21.4)	3.66 (2.51–4.81)	86.6 (76.7–96.5)

### Rapamycin administration resulted in both tumor and PBMC modulation of mTOR targets

**Figure 4:**
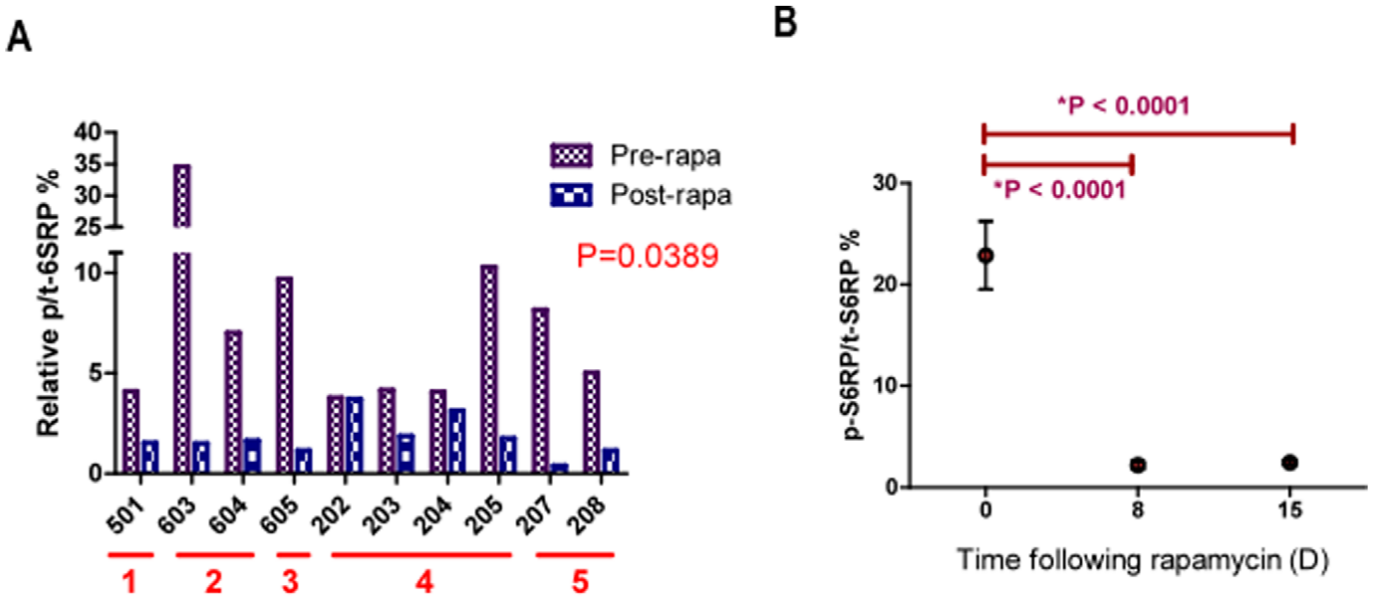
Rapamycin therapy inhibits tumoral and PBMC downstream targets of mTOR in a clinical setting. Modulation of mTOR pathway targets were evaluated in matched tumor (A.) and PBMC samples (B.) to compare pS6RP pre- and post-rapamycin therapy. Electrochemiluminescence (ECL) was utilized to accurately quantify phospho-protein status in tumor and PBMC. Quality control assessments defined 10 tumor and 8 PBMC samples eligible for evaluation. **A**. The red bars below the x-axis indicate patient dosing cohorts. Pre-treatment bars (purple) represent p-S6RP tumor levels prior to rapamycin dosing and post-treatment bars (blue) represent Day 8 levels at tumor surgical excision. Rapamycin led to >2-fold inhibition of tumoral p-S6RP in 8/10 dogs (**A**, p<0.0001). **B**. PBMC phosphorylation of S6RP was significantly inhibited in 8/8 dogs evaluated at Day 8 after rapamycin therapy and was maintained through Day 15 (7 days after the cessation of rapamycin therapy) (**B**, p<0.0001). Matched PBMC and tumor sample data were concordant. Marked post-treatment mTOR pathway inhibition was seen in dogs from all dose cohorts, including the lowest dose cohorts (0.01–0.02 mg/kg), proving that p-S6RP is a very sensitive biomarker of rapamycin administration.

Modulation of mTOR pathway targets were evaluated in matched tumor (**Figure 4A** **.**) and PBMC samples (**Figure 4B** **.**) to compare p-S6RP and p-AKT expression pre- and post-rapamycin treatment. ECL techniques were utilized to accurately quantify the relative percentage of phospho-protein over total protein (p/t) in tumor and PBMC. Quality control assessments defined 10 tumor and 8 PBMC samples eligible for evaluation. Rapamycin led to >2-fold inhibition of tumoral p/t-S6RP (S240/S244) in 8/10 dogs (**Figure 4A** **.**, p = 0.039). PBMC p/t-S6RP inhibition was highly significant at Day 8 and was maintained through Day 15 (n = 8, **Figure 4B** **.**, p<0.0001). Modulation of mTOR pathway targets in PBMC and tumor samples were concordant. Interestingly, marked post-treatment mTOR pathway inhibition was seen in dogs from all dose cohorts, including the lowest dose cohorts (0.01–0.02 mg/kg). It is unlikely that exposures generated from rapamycin treatment in these lowest dose cohorts are clinically active in patients. There was no relationship observed between pharmacokinetic parameters (AUC_0–48hr_, trough concentration, or C_max_) and decrease in pS6RP/t-S6RP in PBMC or tumor (data not shown). This suggests that pS6RP is a highly sensitive biomarker of any rapamycin exposure, but not likely a biomarker of effective exposure or a likely predictor of future clinical response.

### Lack of p/t-AKT up-regulation in rapamycin treated tumors

**Figure 5:**
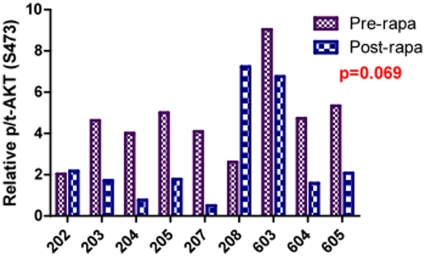
Tumoral AKT phosphorylation is unchanged after short-term exposure to rapamycin in dogs with osteosarcoma. AKT is an important pro-survival pathway in a variety of tumor types. In 9 tumor samples that passed quality control standards there was no significant (p = 0.069) post treatment up-regulation of pAKT (measured by ECL) after 8 days of exposure to rapamycin.

AKT is an important pro-survival pathway in a variety of tumor types. Up-regulation of p-AKT has been suggested to be a consequence of mTOR inhibition. In 9 tumor samples that passed quality control assessments for ECL there was no post treatment up-regulation of relative p/tAKT (S473) after 8 days of exposure to rapamycin (p = 0.069). (**Figure 5**) The influence of longer-term rapamycin exposure on this pathway is unknown.

## Discussion

Results of this study demonstrate that rapamycin can be administered to dogs at pharmacokinetic exposures that are safe and translationally relevant (i.e. achievable in human cancer patients). These exposures modulate the proximate targets of the mTOR pathway in canine tumors and PBMC without inducing AKT phosphorylation. Inhibition of S6RP phosphorylation was a highly sensitive marker of exposure to rapamycin, even in the lowest dose cohorts, such that its pharmacodynamic modulation was not dose dependent.

The biological behavior and histological features of canine and human osteosarcoma are indistinguishable [25], [35]. Both cancers represent primary cancers of bone, occurring primarily in the appendicular skeleton. Most importantly, cancers in both species are characterized by metastasis. Despite complete surgical control of the primary tumor, metastasis to the lungs is the most common cause of death in human and canine osteosarcoma [35]. Recent studies in our laboratory have extended the observation of biological similarities through gene expression analysis of both canine and human cancers [36]. The relevance of osteosarcoma as a model for a highly metastatic human cancer, along with preclinical data generated herein that supports the opportunity to evaluate mTOR inhibitors in canine osteosarcoma, paves the way for clinical evaluation of rapamycin and other mTOR inhibitors in tumor bearing dogs as part of an integrated and comparative drug development approach.

Many questions remain regarding the optimal use of mTOR inhibitors in cancer and in pediatric osteosarcoma in particular. Responses in clinical trials using mTOR inhibitors have been sporadic and not necessarily predicted by cancer histology. Furthermore modulation of traditional PD biomarkers such as phospho-S6RP, as found in this study, is unlikely to define clinically relevant exposures of rapamycin or rapalogs. This finding was supported by recent PK-PD evaluations in human patients with solid tumors that found phosphorylation of S6RP in skin surrogates did not correlate with rapamycin dose or response [37]. Definitive predictive biomarkers of response have yet to be described and are required for successful development of this class of compounds. Acquiring matched PK and PD (tumor and surrogate PBMC measurements) for all dosing cohorts in this study demonstrates a unique attribute of the comparative approach that can aid in the discovery and validation of novel biomarkers that may predict response to mTOR inhibitors. Elaborating on exposure-dose-PD associations will be the focus of future modeling of rapamycin and rapalogs development.

As an immunomodulator, concerns about the impact of rapamycin on regulatory T cells (Tregs) and other negative effectors of the immune system are of unique concern for cancer patients. In this short-term study of rapamycin exposure in dogs with osteosarcoma, there was no evident lymphopenia, increased post-operative infection rate, nor surgical incision healing delays reported. However it is likely that longer-term exposure would be necessary to cause clinical immunosuppression. Additionally, an optimal schedule for long-term exposures to rapamycin in cancer patients must be characterized. Schedules for rapamycin in the setting of immunosuppressive transplantation are not necessarily ideal and may be deleterious as cancer therapies. Optimization of schedule is considered a priority for future canine osteosarcoma rapamycin studies to assess immunosuppression and to determine what regime is most advantageous. Based on the success of this effort, a follow-up evaluation in dogs with metastatic osteosarcoma was launched. Indeed the study aimed to compare three schedules of rapamycin therapy and evaluate chronic tolerability at a fixed rapamycin dose. Chronic administration of this parenteral formulation was not tolerable. Sterile abscess formation at the site of intramuscular (IM) injection occurred and could not be ameliorated by a change to subcutaneous (SQ) administration. In addition, the long-term stability of the described formulation was problematic, thus objectives to compare immune function, evaluate PBMC AKT-mTOR axis modulation and determine if rapamycin treatment is active in dogs with measurable metastatic disease was impracticable. This parenteral formulation has been abandoned in favor of oral dosing for schedule selection studies in dogs. Recent anecdotal evidence has shown tolerability to oral rapamycin dosing in dogs with cancer. Formal PK and tolerability assessments with oral rapamycin formulations in dogs are currently underway.

Rapamycin is the first generation agent in this class of compounds. Rapalogs were developed to improve its solubility. In fact, most are metabolized to rapamycin for their active form. Beyond routes of administration and potency, is very unlikely that the biology of rapamycin and rapalogs will be different. As such these data can be translated to the evaluation of rapalogs in dogs. That being said each new agent is a discrete drug and validation studies will be needed to confirm their safety in dogs with cancer. Additionally, the next phase mTOR kinase inhibitors are likely distinct agents with some but not completely overlapping biologies. Novel studies in dogs may elucidate these unique mechanisms of action and also inform their development.

This work validates dogs with cancer to be relevant models in researching rapalog drug development. Accelerated completion and contemporaneous reporting of tumor bearing dog trials seizes upon the timely opportunity to guide pivotal trial initiation in pediatric sarcoma patients. Future randomized control studies of rapamycin combined with chemotherapy in dogs with osteosarcoma can inform our understanding of how best to use rapalogs in microscopic disease, their most likely efficacious setting. By answering critical adjuvant questions in the dog, results will influence the design of planned Children's Oncology Group (COG) Phase III trials of rapamycin in combinational regimes. Comparative oncology models may also allow further elucidation of PK-PD relationships and provide imaging surrogates effective for early response evaluation in this class of compounds. Collectively these data will be integrated within the development consideration of rapamycin and rapalogs for both canine and human pediatric sarcoma and solid tumor patients.

## Materials and Methods

### Comparative Oncology Trials Consortium

The goals and infrastructure of the COTC have been recently described [24], [25]. This is the second clinical trial in dogs with cancer conducted through this multi-institutional consortium. All COTC trial data were reported electronically and contemporaneously reviewed through a modified form of Oracle Clinical, known as the Cancer Central Clinical Database (C3D), developed through the NCI's Center for Cancer Research (CCR) and Cancer Bioinformatics Grid (CaBIG) and modified for use in canine clinical trials [38].

### Cell lines and in vitro inhibition with rapamycin

#### Immunoblots

Tumor cell lines (BW, SK-primary canine osteosarcoma cell lines, Hong, SH, personal communication; and MCF7, MDA231-human breast carcinoma; American Type Culture Collection) were treated with (100nM for 4 hours) or without rapamycin as described, then lysed in MSD lysis buffer (see below) with complete lysis buffer with protease and phosphatase inhibitors. Protein concentration of lysates was determined by BCA protein assay (Pierce, Thermo Fischer Scientific, Rockford, IL). Lysates were separated with Invitrogen NuPage gels (Invitrogen, Carlsbad, CA) and transferred to nitrocellulose membranes. The blots were probed for the proteins of interest with specific antibodies followed by a secondary antibody (Cell Signaling Technology, Inc, Danvers, MA) and then incubated with SuperSignal chemiluminescence substrate (Pierce). The blots were then exposed to Kodak Biomax Light Film (Kodak, Rochester, NY). The antibodies against AKT and p-AKT (Ser473), S6RP and p-S6RP, and actin were obtained from Cell Signaling.

### Trial eligibility and enrollment

Client-owned pet dogs with histologically confirmed, localized appendicular osteosarcoma, favorable performance status (grade 0 or 1 modified ECOG performance status), and informed owner consent were eligible for enrollment. Eligibility criteria required a measurable tumor amenable to incisional biopsy and surgical resection, and a 72-hour washout from any previous non-steroidal anti-inflammatory drug (NSAID) administration. Physical examination, laboratory [complete blood count (CBC), serum biochemical profile, urinalysis (UA)], and imaging studies were performed to evaluate eligibility prior to enrollment. Exclusion criteria removed dogs weighing less than 15 kg, those with significant co-morbidities (such as renal, liver, and heart failure or coagulopathy), history of inflammatory bowel disease, chronic gastroenteritis, or concurrent chemotherapy, radiation therapy, or biological therapy. Tumor staging included thoracic radiographs performed prior to enrollment. All dogs were evaluated uniformly and treated within a defined clinical protocol with Institutional Animal Care and Use Committee (IACUC) approval at each COTC enrollment site (Colorado State University, University of Illinois, Ohio State University and University of Wisconsin-Madison). The NCI-Comparative Oncology Program (COP) reviewed the eligibility screening and approved trial entry of each patient.

### Rapamycin administration, monitoring, and safety assessment

Dogs underwent a complete physical examination, CBC, serum biochemical profile, UA and pre-treatment biopsies at Day 0. Vital signs (core temperature, pulse, respiratory rate) were recorded at baseline. Dogs received parenteral (IM) rapamycin initially on Day 0 in the early morning and remained hospitalized for serial serum/plasma and whole blood collections over a 48-hour period. Dogs were discharged into the care of their owners and subsequently received rapamycin IM at home via owner administration for 7 consecutive days (once daily). Owners completed an *Owner Assessment Form* on Days 0, 8 and 15 to record impressions of their dog's clinical status throughout the study period.

Definition of acute and chronic toxicities of single and multiple doses of rapamycin was a major goal of this study. Blood samples were collected to define hematologic and biochemical DLT. CBC, biochemical profile and urinalysis were evaluated at Day 0, and then weekly (pre-operatively at Day 8 and Day 15) to define safety. The Veterinary Cooperative Oncology Group Common Toxicity Criteria for Adverse Events (VCOG-CTCAE) was used to determine DLT, defined as any grade 3 or grade 4 (hematologic or non-hematologic) events [32]. DLT toxicity in 1/3 dogs in a cohort (33%) necessitated cohort expansion to ensure tolerability. MTD was defined as one dose level below the maximum achieved in dose-escalation. Any and all adverse events were collected within the electronic database reporting system (C3D) following strict one-week reporting timelines.

### Rapamycin formulation

Rapamycin (R-5000, MW = 914.17; C_51_H_79_NO_13_, >99% pure) was purchased from LC Laboratories (www.lclabs.com, Woburn, MA). The University of Arizona College of Pharmacy (SY) formulated a parenteral rapamycin solution (2 mg/ml) via the recipe in **Table 5**. The pH of the final formulation was 5.09 and density 1.01 g/ml. The formulation was filtered through a 250ml 0.2µm sterile vacuum filter system (Corning Inc). HPLC analysis was used to verify drug concentration using a standard curve of rapamycin spike in concentrations (0.05, 0.1, 0.25, 0.5 mg/ml). Stability studies consisted of HPLC analysis at time zero (t = 0) and after 1-month storage at different temperatures. Stability was maintained at 4 C for 1 month. A portion of the remaining volume was held at 4°C for 6-month stability studies, again showing stability of this initial (COTC003) formulation at 4 C. Although some precipitation was observed due to low solubility, it was overcome with sonication. Total of 3 containers, each containing 250 ml, 250 ml and 200 ml respectively (total of 750 ml), were shipped to the NCI-COP for distribution to COTC sites. A 5 ml aliquot (before filtration) was shipped separately for endotoxin testing. Endotoxin was measured prior to rapamycin patient use, using Limulus Amebocyte Lysate QCL-1000® kits (Cambrex, Inc., Watersville, MD) and results showed negligible levels.

**Table 5:** Rapamycin Formulation.

Ingredients	Formula	Ideal Amount	Actual Amount
Rapamycin	1800.0 mg	1800.0 mg	1801.49 mg
Ethanol	10.0%	90.0 ml	90.0 ml
Propylene glycol	40.0%	360.0 ml	360.0 ml
Benzyl alcohol	1.5%	13.5 ml	13.5 ml
Benzoate buffer pH 4.05	5.0%	45.0 ml	45.0 ml
Total Volume q.s. WFI	900.0 ml	900.0 ml	900.0 ml
**Final Concentration**	2.0 mg/ml	2.0 mg/ml	2.002 mg/ml

### Reformulation for chronic dose study

The formulation for rapamycin was augmented for the chronic dose study (Dr. Samuel Yalkowsky, University of Arizona College of Pharmacy) from the original recipe used in COTC003 (described above). In this new formulation, additional benzoic acid buffer (total of 5% w/v) was added and heat used to ensure rapamycin dissolution. Post hoc stability assessments in two laboratories (SY and DG) revealed degradation of the parent drug. Chromatographs showed two resultant peaks: the first consistent with rapamycin (RT = 0.55–0.56 min) and a second unknown peak (RT = 0.96–0.99 min) (via DS SCIEX 3200 Q-TRAP LC/MS/MS system equipped with a HPLC column (DG)). These were repeated for verification with the same result.

### Rapamycin Pharmacokinetic Sampling

Serial whole blood (5 ml, with K_3_ EDTA anticoagulant) samples were obtained by venipuncture prior to and 0.5, 1, 2, 6, 24 and 48 hours after the first dose of rapamycin. The second dose of rapamycin was administered after the 48-hour sample was obtained. Subsequent doses were administered on days 3–8. Additional pharmacokinetic samples were obtained on days 8 (192h, day of definitive resection) and 9 (hr 216, after last day of dosing) and 15 (360 hr). When applicable, all whole blood collections were obtained at trough prior to the next scheduled rapamycin administration. Whole blood was transferred to cyrovials and stored at −80°C until analysis. Whole blood, serum and plasma were collected from all patients. Serum and plasma samples were stored for post-hoc hypothesis generating assessments.

### Rapamycin Pharmacokinetic Assay and Analysis

A method for determining rapamycin concentrations in dog whole blood was developed by Covance, Inc (Covance Bioanalytical Services, LLC, Indianapolis, IN) based on a previous protocol for human whole blood rapamycin pharmacokinetics (Covance 2100-358). Rapamycin was extracted from dog whole blood by protein precipitation followed by solid-phase extraction. The eluate was analyzed using HPLC with MS/MS detection. Rapamycin, the internal standard (ISTD), tacrolimus, and normal canine whole blood were used for calibration and quality controls. The standard curve range for rapamycin is from 0.250 to 50.0 ng/mL, using a whole blood sample volume of 0.200 mL. Clinical sample results were calculated using peak area ratios and calibration curves were generated using a weighted (1/x^2^) linear least-squares regression. The calibration standards and quality control samples were within acceptance criteria and the assay method validated with repeatable precision and accuracy. All data were acquired, processed, and reported using Applied Biosystems/MDS-Sciex Analyst Version 1.4 software (www.lifetechnologies.com, Carlsbad, CA).

Rapamycin concentration-time data were analyzed using non-compartmental methods. The peak rapamycin concentration (C_max)_) and time to peak concentration (T_max_) were determined from concentration-time plot of each subject's data. Area under the concentration time curve to the time point measured at 48hr after the first dose (AUC_0–48h_) was calculated with the linear trapezoidal method. The terminal rate constant was derived from the slope of the natural log transformed concentrations and times on the terminal elimination phase of the decay curve. Terminal elimination half-life was calculated by dividing 0.693 by the terminal rate constant. The relationship of pharmacokinetic parameters and pharmacodynamic variables were examined using scatter plots.

### Tumor collections

Serial biopsies were required from all dogs to evaluate mTOR target pharmacodynamics in tumors at baseline and their modulation following rapamycin therapy. Biopsy techniques were prospectively defined by standard operating procedures (SOPs) applied uniformly at all participating COTC sites. Incisional pre-treatment biopsies were collected (11 gauge Jamshidi) before rapamycin administration, with three (3) samples obtained at various planes within the tumor to capture natural disease heterogeneity. Each of these sections were divided equally and one half fixed in 10% formalin and the other half flash frozen in liquid nitrogen. Post treatment samples were obtained at surgical excision of the tumor via standard techniques/amputation with biopsies collected within 20 minutes of limb removal. Again, three (3) sections of the tumor were sampled at various angles/planes, divided and stored as above. After the 3 sections were taken the whole tumor was resected from the limb and divided into two (2) equal portions. Half of the resected tumor was submitted for standard histopathologic evaluation at the enrolling COTC institution, and the other half subdivided into two equal portions for formalin fixation and flash freezing. After 24 hours of fixation formalin samples were transferred to 80% ethanol and stored at 4 C. Frozen samples were stored at −80 C. All tissue samples were shipped to the NCI-COP at end of the study for batch evaluation.

### PBMC Collections

PBMC collections were used for the assessment of correlative PD endpoints (mTOR and down stream pathway inhibition after rapamycin treatment). PBMCS were collected in two (2) 8 ml BD CPT vacutainers (Becton Dickinson, Franklin Lakes, NJ) with sodium heparin, each filled completely with whole blood. They were centrifuged for one hour, the aqueous portion transferred into 15 ml conical tubes, and shipped at room temperature to the NCI-COP on the same day as their collection.

Protein lysates were made from these samples. Samples were brought up to a volume of 15mL using 1× PBS. A cell pellet was obtained by centrifuging for 15 minutes at 300 rcf (around 1200–1500 rpm). Supernatant was aspirated without disturbing the cell pellet. To lyse the red blood cells, 500uL of DEPC treated water was added to the pellet and pipetted up and down five times. Then 9.5mL of 1× PBS was quickly added. Samples were centrifuged for 10 minutes at 300 rcf and supernatant was aspirated. If red blood cells could be seen at this point, the process of lysing red blood cells was repeated. Once a white pellet was isolated, 200uL of 1× PBS was added and cells were transferred to an Eppendorf tube and stored at −80°C for future ECL analysis.

### Pharmacodynamic Assessments

#### Electrochemiluminescence assays

The samples (cell lines, tumor, PBMC) used for ECL were prepared based on the Meso-Scale Discovery (MSD) lysate preparation protocol (Meso-Scale Discovery, Gaithersburg, MD) using complete lysis buffer with protease and phosphatase inhibitors. For quantitative analysis of phospho-proteins, duplex t/p-AKT (S473) and duplex t/p-S6RP (S240/S244) were obtained from MSD, used following manufacturer's instructions, and read with Sector Imager 2400 (MSD). A total of 25 ug of lysates were used per well for the duplex assays. Out of the 19 paired tumor biopsies analyzed, 9 were excluded due to values below the detection limit, of which 8 were from one trial site. Large variation (100-fold) was seen with total S6RP in biopsies therefore the ratio between p-S6RP/t-S6RP was used for more appropriate statistical analysis. PBMC from 8 patients were analyzed for p-S6RP/t-S6RP and all data was shown.

### Histopathology Review

Formalin fixed tumor biopsies were paraffin embedded in blocks, sectioned and stained with hematoxylin and eosin. A single veterinary pathologist (TO) reviewed all samples for tumor integrity and presence of necrosis. This analysis guided matched frozen sample selection for ECL.

## Supporting Information

Figure S1 Validation of rapamycin-mediated inhibition of p-S6RP and quantitative electrochemiluminescence assays in canine osteosarcoma A. Immunoblot shows rapamycin-mediated (100 nM) inhibition of p-S6RP in two canine osterosarcoma cell lines (BM, SK) and human breast cancer cell lines (MCF7 and MDA231). Controls were Jurkat cells treated with LY (−) or PMA (+). B. Quantitative determination of total and p-S6RP (S240/244) with electrochemiluminescence (ECL) assay. C. Quantification of ECL results illustrate that treatment with rapamycin results in approximately 50× reduction of p/t-S6RP in two canine osteosarcoma cell lines (BW and SK; p/t-S6RP Rapa %) compared to untreated controls (BW and SK; p/t-S6RP ctrl %).
(1.56 MB TIF)
