# Supplementary figures and images for: Rapamycin Pharmacokinetic and Pharmacodynamic Relationships in Osteosarcoma: A Comparative Oncology Study in Dogs
Source: PLoS One. 2010 Jun 8;5(6):e11013. doi: 10.1371/journal.pone.0011013 (PMC2882366; doi:10.1371/journal.pone.0011013)

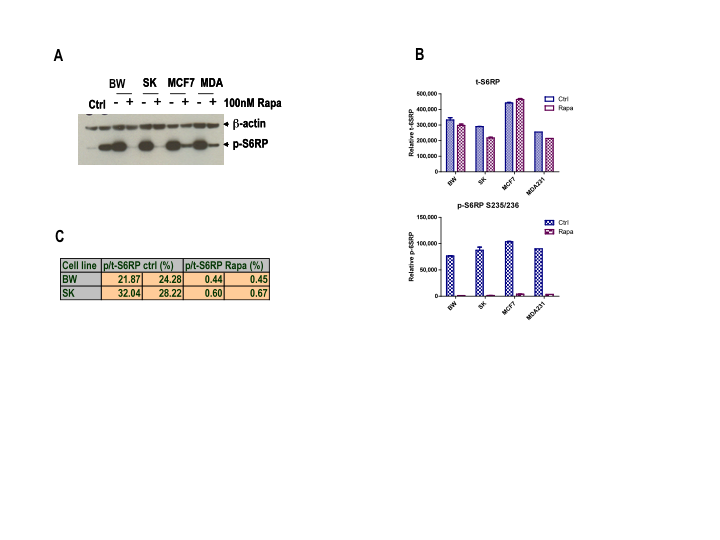

Supplement: Figure S1 — Validation of rapamycin-mediated inhibition of p-S6RP and quantitative electrochemiluminescence assays in canine osteosarcoma A. Immunoblot shows rapamycin-mediated (100 nM) inhibition of p-S6RP in two canine osterosarcoma cell lines (BM, SK) and human breast cancer cell lines (MCF7 and MDA231). Controls were Jurkat cells treated with LY (−) or PMA (+). B. Quantitative determination of total and p-S6RP (S240/244) with electrochemiluminescence (ECL) assay. C. Quantification of ECL results illustrate that treatment with rapamycin results in approximately 50× reduction of p/t-S6RP in two canine osteosarcoma cell lines (BW and SK; p/t-S6RP Rapa %) compared to untreated controls (BW and SK; p/t-S6RP ctrl %). (1.56 MB TIF) [file pone.0011013.s001.tif]
